# 

**Published:** 1873-12

**Authors:** 


### CONTENTS:

ORIGINAL COMMUNICATIONS:

ARTICLE I. Lanfranc of Milan. His Brief. Reduced to our Vulgar Phrase. By Jas. N. Hyde, M.D., Chicago.... 705

ART. II. The Premonitory Symptoms of Insanity. By H. R. Bigelow, Boston.... 717

ART. III. Abstract of the Discussion in the Pathological Society, London, June and July, 1873, on the Anatomical Relations of Pulmonary Phthisis to Tubercle, with Remarks. By Z. Collins McElroy, Zanesville, O....... 723

ART. IV. Transactions of Chicago Society of Physicians and Surgeons. Meeting Nov. 10, 1873. Reported by P. S. Hayes, M.D.... 742

SELECTIONS:

A Case of General Paresis. By J. B. Stonehouse, M.D.... 743

EDITORS' BOOK TABLE.... 748

EDITORIAL.... 753

REPORTS OF SOCIETIES.... 760

OBITUARY—W. R. Marsh, M.D.... 761

